# Bone Formation in Pleomorphic Adenoma: A Case Report

**DOI:** 10.7759/cureus.22868

**Published:** 2022-03-05

**Authors:** EB Robson Gubod, Anand Ramanathan, Sherrie Chong Mei Yee, Wanninayake M Tilakaratne

**Affiliations:** 1 Department of Oral & Maxillofacial Clinical Sciences, Faculty of Dentistry, Universiti Malaya, Kuala Lumpur, MYS; 2 Oral Cancer Research & Coordinating Centre, Faculty of Dentistry, Universiti Malaya, Kuala Lumpur, MYS; 3 Oral & Maxillofacial Surgery Department, Hospital Tengku Ampuan Rahimah, Klang, MYS

**Keywords:** chondroid stroma, osseous stroma, bone, salivary gland tumour, benign, pleomorphic adenoma

## Abstract

Pleomorphic adenoma is a benign tumour with variable cytomorphological and architectural elements. It is the most common salivary gland tumour in children and adults. We report a case of a 32-year-old Malay woman who presented with a slow-growing, painless, firm, and mobile nodule of 1 cm x 1 cm in size at the angle of her mandible on the right side which could be palpated bimanually. Intraorally, this nodule could be palpated at the retromolar area. On excisional biopsy, this nodule was reported as a pleomorphic adenoma with predominant chondroid stromal elements and a large area of bone formation in the stroma. We discuss the possible pathogenesis, differential diagnosis, and clinical significance of this exceptionally rare phenomenon of bone formation in pleomorphic adenoma in minor salivary glands with the literature review. The clinical significance and relevance on treatment outcome for pleomorphic adenoma with bone formation is currently uncertain as there are only a few cases reported in the literature. A good follow-up study is recommended to assess the clinical significance of pleomorphic adenoma with extensive bone formation.

## Introduction

Pleomorphic adenoma (PA) is a benign tumour with variable cytomorphological and architectural manifestations [1]. PA was first termed by Willis [2]. It is also known as a benign mixed tumour, and is the most common salivary gland tumour in children and adults [1]. In the early days, PA was also referred to as a mixed tumour, enclavoma, branchioma, endothelioma, and enchondroma [3]. PA is most commonly found to occur among adult females in the third to fifth decade of life [2]. This tumor commonly occurs in the parotid gland (85%), followed by minor salivary glands and submandibular glands (10% and 5% respectively) [2]. PA usually appears as a slowly enlarging mass, painless, and not involving the facial nerve [4]. The tumour is made of cellular and mesenchymal components [2]. The cellular components of PA are mainly comprised of epithelial and myoepithelial cells [5]. The diagnosis of PA is based on the identification of these components [2]. We present a case of PA of the right angle of the mandible region with extensive bone formation.

## Case presentation

A 32-year-old Malay woman presented with painless swelling over the right angle of the mandible which was slowly growing in size. She noticed the swelling about two years ago which was small but increased to its current size over the years. There was no history of toothache and gum swelling on the right side of the mandible. Her past medical history and family history was non-contributory. She did not have any risk habits. Extraoral examination showed that the face was slightly asymmetrical on the right side. Her mouth opening and temporomandibular joints were normal. There was no pain on any of her muscles of mastication. There was a firm and non-tender nodular swelling over the right angle of the mandible. This swelling had smooth margins and was mobile, not fixed to skin or surrounding structure. The nodule was palpable bimanually. This swelling measured about 1cm x 1cm in size. On intraoral examination, the swelling was palpated at the retromolar area. She did not have any dental caries, filled teeth, or missing teeth, but had partially erupted upper right canine and medially displaced upper right first molar. Her gingiva was healthy. The dental panoramic tomography (DPT) did not show any pathological lesions at the angle of the mandible on the right side. Thus, the swelling on her right angle of the mandible was considered clinically benign and an excisional biopsy was carried out under local anaesthesia. Macroscopically, the specimen was a soft tissue nodule measuring about 0.7 x 0.7 cm in size. The nodule was well-circumscribed and had firm to hard consistency. The cut surface of the nodule was light tan in colour. 

**Figure 1:**
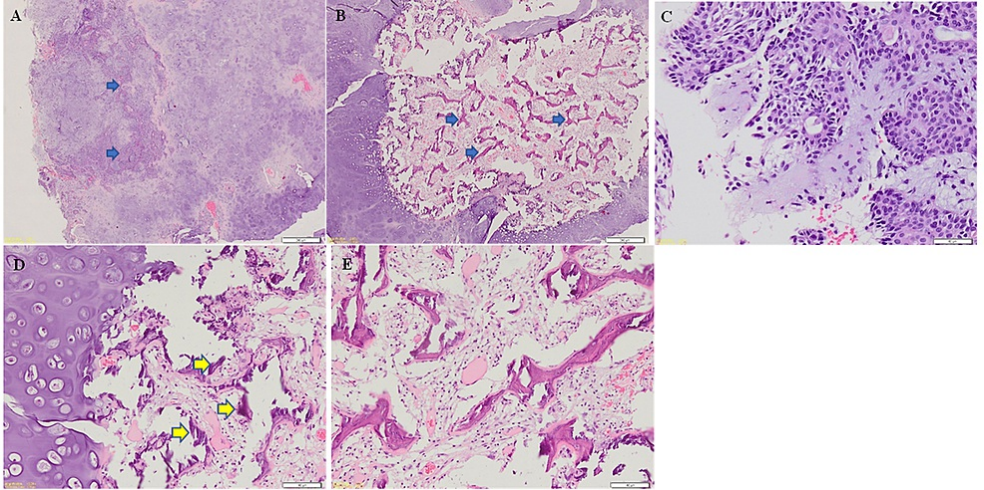
Photomicrographs shows (A) sheets of chondroid stroma with islands of myoepithelial cells (arrows) (Original magnification: 2x; Stain: H&E), (B) area with thin trabeculae of woven bone (arrows) (Original magnification: 2x; Stain: H&E), (C) tumour cells composed of epithelial cells and myoepithelial cells showing ductal formation (Original magnification: 20x; Stain: H&E), (D) chondroid stroma with chondrocytes residing within lacunae and spicules of woven bone formation (arrows) (Original magnification: 10x; Stain: H&E). (E) trabeculae of woven bone with surrounding fibro-fatty marrow (Original magnification: 10x; Stain: H&E).

The histopathological examination confirmed the diagnosis of pleomorphic adenoma. The haematoxylin and eosin sections showed that the lesion was encapsulated and composed of predominantly chondroid stroma with chondrocytes residing in the lacunae, and minimal areas of hyalinization (Figures 1A, 1B, and 1D). The tumour cells were arranged in islands and nests and formed ductal structures containing eosinophilic materials in the lumen (Figure 1C). The tumour cells were composed of plasmacytoid cells with uniform oval to round nuclei and eosinophilic cytoplasm. A large area with trabeculae and cords of metaplastic woven bone formation was present (Figures 1B, 1D, and 1E). These bone formations were present within a myxoid vascular stroma, adjacent to the chondroid area. The bone exhibits areas of irregular peripheries and osteocytes within lacunae. Osteoblastic rimming was also seen in a few areas. Two weeks after the excisional biopsy, the healing at the biopsy site was satisfactory. However, the patient did not turn up for her following review. 

## Discussion

PA most often occurs in the third to sixth decades of life, but it has been reported to occur in all ages, with an average age at presentation being 45 years [1]. PA has site predilection for the parotid gland, and other sites that are commonly affected are the submandibular gland and palate [1]. It is usually solitary, but metachronous and synchronous tumours may also occur rarely [1].

Histologically, the tumour is composed of variable epithelial, myoepithelial, and stromal components in a mixture of histological patterns. The cellular elements may show a spectrum of phenotypes which include oval, spindled, epithelioid, clear, and plasmacytoid morphology [1]. However, the identification of epithelial and myoepithelial components is essential for the diagnosis of PA [1].

The stromal elements can vary from myxoid, lipomatous, chondromatous, hyalinized, to osseous in nature [1]. The most frequent stromal elements appear to be myxomatous and chondroid areas [6]. Bone formation in PA is a rare phenomenon. There were only a limited number of reported PAs with predominant bone formation [6]. 

This rare phenomenon is most commonly seen in a parotid lesion and exceptionally rare in PAs arising in minor salivary glands [7]. There were only a limited number of reported PAs with predominant bone formation [6]. Shigeishi et al [6] reported a case of PA with bone tissue formed within chondroid tissue where the mineralized chondroid tissue merged continuously into osteoid tissues as seen in the present case. They also noted degenerated cartilage cells in the mineralized cartilage. The bony component was composed of mature bone trabeculae with abundant lacunae containing osteocytes. Moreover, the author also suggested the possibility of endochondral ossification as they observed that the bone tissue seemed to be formed within areas of chondroid tissues as observed in the present case. A recent study on 21 cases of minor salivary gland PA reported that bone formation was seen only in 3 cases (14.3%) [8]. 

A case report of a mixed tumour of the submandibular gland showed trabecula of bone formation within chondroid nodules. They suggested that bone tissue formation was by endochondral ossifications [9]. However, in contrast, in another case report of an ossifying PA of the maxillary antrum, the authors suggested that the source of the bone matrix was from metaplastic myoepithelial cells rather than endochondral ossifications [10]. There was another case reported describing a case of PA of the upper lip with bone formation, where the bone tissue was formed by direct deposition of osteoid tissue by myoepithelial cells and also by partial endochondral ossification [11]. Myxoid stroma is associated with a high recurrence rate after surgical removal [12], while PAs with prominent hyalinization are more likely to undergo malignant transformation [13]. However, clinical significance of bone formation in pleomorphic adenoma is still undetermined as it is an exceptionally rare phenomenon and very few had been reported in the literature.

In the current case, the bone was composed of woven bone and located adjacent to chondroid areas. Histologically, compared to the cases previously reported in the literature, the features are more or less the same. Most cases are associated with the presence of chondroid nodules suggesting a possibility of endochondral ossification.

The differential diagnosis for this case includes benign tumours of chondroid origin such as chondromyxoid fibroma (CMF). CMF is a benign chondroid neoplasm that accounts for less than 1% of all cartilaginous tumour, and rarely occurs in the head and neck [14]. Patients often present with tinnitus headaches, pain, hearing loss, vertigo, visual disturbances, and sinonasal congestion depending on the sites involved [14]. Microscopically, CMF consists of a lobular proliferation of uniform spindle or stellate cells with prominent eosinophilic cytoplasm with a myxoid background [14]. The presence of ductal elements in PA can help distinguishing it from CMF [14].

Extraskeletal chondroma, also known as chondroma of soft parts is another differential diagnosis. It is a benign tumour composed of predominantly hyaline cartilage with no connection to bone or periosteum [15]. It is usually small, measuring less than 3cm in diameter [15]. Chondroma appears as a well-circumscribed, lobular mass of mature hyaline cartilage demonstrating well-formed lacunae containing small chondrocytes with pale cytoplasm and small, round nuclei [16]. This entity is difficult to differentiate from low-grade chondrosarcoma [16]. However, ductal elements are not found in this tumour, which distinguishes it from PA [16].

Other differential diagnoses are benign tumours of the bone which includes osteoma, osteoid osteoma, and osteoblastoma. Osteoma is a benign neoplasm of the bone composed of mature lamellar bone having compact bone or trabecular bone or a combination of both [17]. Osteoblastoma is a benign bone-forming tumour which is greater than 20 mm in size with prominent osteoblastic rimming. Clinically they are asymptomatic. They contain haphazardly arranged mineralized trabeculae of bone with cellular fibrous stroma. Some of the bones are haematoxophilic giving them a blue-bone appearance [18]. On the other hand, osteoid osteomas are benign bone-forming neoplasms characterized by limited growth potential which are less than 20 mm in size and have histopathological features similar to osteoblastoma. However, clinically osteoid osteoma presents with severe pain [19]. 

## Conclusions

PA can present with various stromal features with the most common being the myxoid, chondroid, myxochondroid, and fibrous stroma. However, osseous stroma formation in PA is a rare occurrence. The clinical significance and relevance on treatment outcome for PA with bone formation is currently uncertain as there are only a few cases reported in the literature. A good follow-up study is recommended to assess the clinical significance of PA with extensive bone formation.
